# Short-term results of curcumin, quercetin, hyaluronic acid and chondroitin sulfate (Ialuril Soft Gels®) in the management of chronic prostatitis/primary prostate pain syndrome: a single-center prospective study

**DOI:** 10.3389/fsurg.2025.1700681

**Published:** 2025-11-10

**Authors:** Valerio Iacovelli, Matteo Vittori, Marco Carilli, Chiara Cipriani, Marta Signoretti, Michele Antonucci, Filomena Petta, Beatrice Filippi, Giulia Di Giovanni, Francesco Maiorino, Filippo Antonino Maria Saccà, Giuseppe Stella, Carlo Brocca, Pierluigi Bove

**Affiliations:** 1 Urology Unit, San Carlo di Nancy General Hospital - GVM Care and Research, Rome, Italy; 2 Minimally Invasive and Robotic Urology Unit, Tor Vergata University Hospital, Rome, Italy; 3 Department of Experimental Medicine, University of Rome Tor Vergata, Rome, Italy; 4 Department of Surgical Sciences, University of Rome Tor Vergata, Rome, Italy

**Keywords:** curcumin, quercetin, hyaluronic acid, chondroitin sulfate, CP/PPPS, prostatitis

## Abstract

**Aim of the study:**

Management of Chronic Prostatitis/Primary Prostate Pain Syndrome (CP/PPPS) is challenging and characterized by conflicting results. This study aimed to evaluate the efficacy of a food supplement based on Curcumin, Quercetin, Hyaluronic Acid and Chondroitin Sulfate (Ialuril Soft Gels®) in CP/PPPS treatment.

**Materials and methods:**

Data of consecutive male patients who referred to our Institution for CP/PPPS were prospectively collected between Oct-2022 and Jan-2023. Patients with maximum flow rate <15 ml/s, post-void residual >150 ml and/or previous prostate surgery were excluded. Variables including age, body mass index (BMI), prostate volume (PVol) and serum prostate specific antigen (PSA) were collected. Patients were asked to fulfil standardized questionnaires to assess severity of pain, lower urinary tract symptoms (LUTS) and erectile function, including Symptom Severity Index (SSI), Symptom Frequency Questionnaire (SFQ), National Institutes of Health Chronic Prostatitis Symptom Index (NHI/CPSI) (pain and LUTS domains), International Prostate Symptom Score (IPSS) and Quality of Life score (IPSS-QoL), and International Index of Erectile Function (IIEF-5). Changes in standardized questionnaires were evaluated at baseline, 30- and 90-days after enrolment. Patients were administered 2 gelcaps of Ialuril Soft Gels® (curcumin 200 mg, quercitin 200 mg, hyaluronic acid 100 mg, chondroitin sulfate 200 mg) once a day for 60 days.

**Results:**

Twenty patients were analysed. Baseline features were age [median 50 years (IQR 46–51)], BMI [24.2 (22.8–28.3)], PVol [42 ml (30–52)], PSA [1.0 ng/ml (0.8–1.8)], SSI [54 (27–55)], SFQ [20 (13–26)], NHI/CPSI [pain domain 10 (8–13); LUTS domain 7 (4–8)], IPSS [13 (10–18)], IPSS-QoL [3 (2–3)] and IIEF-5 [17 (14–22)]. SSI and SFQ scores showed statistically significant differences both at 30- [SSI 45 (22–49), *p* < 0.001; SFQ 16 (11–20), *p* = 0.035] and 90-days [SSI 33 (17–42), *p* < 0.001; SFQ 13 (9–16), *p* < 0.001]. IPSS and NHI/CPSI pain and LUTS domains showed statistically significant differences at 30-days [IPSS 12 (10–13), *p* = 0.002; NHI/CPSI pain domain 6 (5–9), *p* < 0.001; NHI/CPSI LUTS domain 7 (2–9), *p* = 0.033, respectively], but not at 90-days (all *p* > 0.05). IPSS-QoL and IIEF-5 scores did not report any statistically significant difference during follow-up (*p* = 0.12 and *p* = 0.23, respectively). No adverse events were recorded.

**Conclusions:**

Ialuril Soft Gels® seems to be effective in reducing the severity of pain and urinary symptoms in CP/PPPS patients at short-term follow-up.

## Introduction

Chronic Prostatitis/Primary Prostate Pain Syndrome (CP/PPPS) (1) is defined by the European Association of Urology (EAU) as a clinical condition characterized by persistent or recurrent episodic prostatic pain and falls under Category III of the National Institutes of Health (NIH) prostatitis classification (2). According to the International Continence Society (ICS) terminology for sexual health in men with lower urinary tract (LUT) and pelvic floor (PF) dysfunction, CP/PPPS should be understood as part of the broader entity known as Chronic Prostatitis/Chronic Pelvic Pain Syndrome (CP/CPPS) (3). As per the EAU definition, CP/PPPS involves pain typically reproducible on prostate palpation, in the absence of proven infection or identifiable local pathology. Although historically referred to as “chronic prostatitis”, this term is increasingly considered inappropriate by expert consensus (1). CP/PPPS is driven by a combination of acute pain mechanisms (e.g., inflammation or infection), chronic central nervous system (CNS) dysfunction, and psychosocial factors (emotional, cognitive, behavioral, and sexual). Neuropathic pain is common and should be assessed (1). CP/PPPS frequently coexists with lower urinary tract symptoms (LUTS), sexual dysfunction, and negative cognitive, emotional, or behavioral consequences, significantly impairing quality of life and presenting therapeutic challenges due to its complex and multifactorial etiology (1–3).

Conventional oral treatments - including alpha-blockers, antibiotics, anti-inflammatories, and phytotherapeutics - often yield unsatisfactory results, underscoring the need for alternative or adjunctive therapies (1). Recent attention has turned to bioactive compounds with anti-inflammatory and antioxidant properties (4). Among these, Curcumin (5) and Quercetin (6, 7) have shown potential in modulating inflammatory pathways, while Hyaluronic Acid and Chondroitin Sulfate are known to contribute to mucosal and epithelial repair, particularly within the urinary tract (8).

This study evaluates the clinical efficacy and safety of Ialuril Soft Gels ®, a food supplement that combines these four agents, in a cohort of patients with CP/PPPS. We hypothesize that this formulation may offer a synergistic benefit in ameliorating the hallmark symptoms of CP/PPPS.

## Materials and methods

This investigation was designed as a prospective study conducted at a single tertiary care academic medical center over a four-month period, from October 2022 to January 2023. The study population comprised consecutive adult male patients (≥18 years of age) who presented with a clinical diagnosis consistent with chronic prostatitis/chronic pelvic pain syndrome (CP/PPPS), as defined by the National Institutes of Health (NIH) diagnostic criteria (2). These criteria include the presence of urogenital pain or discomfort persisting for more than three months in the absence of demonstrable bacterial infection (1–3).

Inclusion criteria were clinical diagnosis of CP/PPPS based on NIH guidelines (i.e., chronic urogenital symptoms lasting longer than three months with no evidence of bacterial infection), provision of informed consent for participation in the study, including agreement to attend follow-up visits and assessments. Exclusion criteria included a maximum urinary flow rate (Qmax) < than 15 ml/s, as measured by uroflowmetry, post-void residual (PVR) urine volume >150 ml, measured via bladder ultrasound, a prior history of surgical interventions involving the prostate gland, current or recent active urinary tract infection (UTI), confirmed through urinalysis and urine culture during the enrollment phase within the 30 days preceding the baseline.

Eligible participants received a daily regimen of Ialuril Soft Gels ®, administered as two gel capsules per day over a 60-day period. Each daily dose contained the following active compounds: Curcumin 200 mg, quercetin 200 mg, hyaluronic Acid 100 mg, chondroitin sulfate: 200 mg.

No additional pharmacological treatments targeting CP/PPPS or BPH/LUTS were prescribed during the study period to avoid confounding effects.

At baseline, demographic and clinical variables were documented, including age, body mass index (BMI), prostate volume (PV, assessed via transrectal ultrasound), and serum prostate-specific antigen (PSA) levels.

Patient-reported outcomes were evaluated using a battery of validated symptom and QoL questionnaires at three time points: baseline (T0), mid-treatment (Day 30, T2), and post-treatment (Day 90, T2). The following instruments were utilized: NIH Chronic Prostatitis Symptom Index (NIH-CPSI), including separate scores for the pain domain and the lower urinary tract symptoms (LUTS) domain; International Prostate Symptom Score (IPSS) and IPSS QoL Subscore (IPSS-QoL), International Index of Erectile Function (IIEF-5), Symptom Severity Index (SSI), Symptom Frequency Questionnaire (SFQ). All the questionnaires were validated in Italian and completed directly by the patients during clinical visits.

In this study, artificial intelligence (AI), specifically ChatGPT (https://www.chatgpt.com), was used solely for reviewing the English language in its grammar, syntax, and style, without affecting content, citations, or interpretative and conclusive discussions.

### Statistical analysis

All continuous variables were summarized using median values and interquartile ranges (IQRs) to account for potential non-normal data distribution. Changes in questionnaire-based scores over the course of the study were analyzed using the Wilcoxon signed-rank test for paired, non-parametric data. A two-tailed *p*-value less than 0.05 was considered indicative of statistical significance. Statistical analyses were conducted using appropriate software tools in accordance with standard biostatistical practice.

## Results

A total of 20 male participants completed the study protocol. The cohort had a median age of 50 years (IQR 46–51), with a average BMI of 24.2 kg/m^2^ (IQR 22.8–28.3). The median prostate volume was 42 ml (IQR 35–51), median Qmax was 17.8 ml/s (IQR 15.5–22) and median PSA level was within normal range at 1.0 ng/ml (IQR 2–0.4).

Patients demonstrated significant and consistent reductions in both the Symptom Severity Index (SSI) and Symptom Frequency Questionnaire (SFQ) over time. SSI scores decreased from a baseline median of 54–45 at 30 days (*p* < 0.001), and further to 33 by 90 days (*p* < 0.001). SFQ scores, which reflect the frequency of bothersome symptoms, improved from 20 at baseline to 16 at 30 days (*p* = 0.035), and 13 at 90 days (*p* < 0.001).

The NIH-CPSI Pain Domain, a direct measure of chronic pelvic pain intensity, showed a substantial improvement. The median pain score dropped from 10 at baseline to 6 at 30 days (*p* < 0.001). Although it slightly increased to 7 at 90 days, this change was not statistically significant (*p* = 0.062), indicating some variability but overall maintenance of pain relief. The LUTS domain of the NIH-CPSI showed a modest decline from 7 to 7 at 30 days (*p* = 0.033), and 6 at 90 days (*p* = 0.071).

The International Prostate Symptom Score (IPSS), a broader measure of urinary symptoms, improved from a baseline of 13–12 at both 30 and 90 days. While statistically significant at 30 days (*p* = 0.002). IPSS QoL scores remained relatively unchanged, moving from a baseline of 3 to 2–3 at follow-up (*p* = 0.12).

IIEF-5 scores, assessing erectile function, remained stable throughout the study (median score: 17 at all time points, *p* = 0.23).

Importantly, the supplement was well tolerated by all patients. No adverse events were recorded during the 90-day follow-up period, underscoring its potential as a safe adjunctive treatment for CP/PPPS (Table 1).

**Table 1:** Baseline characteristics and results of questionnaires and functional outcomes during follow-up.

Questionnaire	Baseline	30 days, *p*-value*	90 days, *p*-value**
SSI	54 (27–55)	45 (22–49) ***p*** **<** **0.001**	33 (17–42) ***p*** **<** **0.001**
SFQ	20 (13–26)	16 (11–20) ***p*** **=** **0.035**	13 (9–16) ***p*** **<** **0.001**
NHI/CPSI	Pain domain 10 (8–13) LUTS domain 7 (4–8)	Pain domain 6 (5–9) ***p*** **<** **0.001** LUTS domain 7 (2–9) ***p*** **=** **0.033**	Pain domain 7 (5–11) *p* = 0.2 LUTS domain 8 (3–9) *p* = 0.33
IPSS	13 (10–18)	12 (10–13) ***p*** **=** **0.002**	11 (10–13) *p* = 0.6
IPSS-QoL	3 (2–3)	3.2 (2–4) *p* = 0.12	3.3 (2–4) *p* = 0.15
IIEF-5	17 (14–22)	18 (13–21) *p* = 0.23	18 (14–22) *p* = 0.3

IPSS, International Prostate Symptom Score; IPSS-QoL, International Prostate Symptom Score-Quality of Life; IIEF 5 International Index of Erectile Function; NHI/CPSI, NIH chronic prostatitis symptom index; SFQ, symptom frequency questionnaire; SSI, symptom severity index.

* *p*-value between 30 days follow-up and baseline.

** *p*-value between 30 and 90 days follow-up.

## Discussion

Primary Prostate Pain Syndrome (PPPS) is characterized by persistent or recurrent pain localized to the prostate region in the absence of identifiable bacterial infection or overt pathology (1). PPPS is often accompanied by lower urinary tract symptoms (LUTS), erectile dysfunction, and considerable psychosocial burden, including anxiety, depression, and diminished QoL (1, 2). Reliable prevalence data for PPPS are limited, as symptom overlap with conditions like benign prostatic enlargement may lead to misclassification (9). Population-based studies report prostatitis-like symptoms in 2.2–14.2% of men (10), with risk increasing with age—men aged 50–59 have a 3.1-fold higher risk than those aged 20–39 (1). Despite decades of research, treatment remains challenging due to its multifactorial etiology and poorly understood pathophysiology.

Historically, the condition has been variably referred to as “chronic nonbacterial prostatitis”, although this nomenclature has largely fallen out of favor (1, 2). Nevertheless, it continues to be clinically equated with PPS due to overlapping symptomatology. Importantly, in a recent Cochrane systematic review, Franco et al. reported that conventional pharmacologic treatments, such as alpha-blockers and antibiotics, have demonstrated limited efficacy, particularly in patients without demonstrable infection or bladder outlet obstruction (11). This has prompted interest in multimodal, non-antibiotic strategies, including nutraceutical and glycosaminoglycan (GAG) replenishment therapies.

The present prospective study contributes to the growing body of literature supporting the use of nutraceutical agents with anti-inflammatory, antioxidant, and urothelial-protective properties in the management of CP/PPPS (12, 13).

This study suggests that a food supplement containing curcumin, quercetin, hyaluronic acid, and chondroitin sulfate (Ialuril Soft Gels ®) may significantly alleviate symptoms of CP/PPPS, especially in terms of pain intensity and symptom frequency. Notably, these improvements were observed as early as 30 days and were sustained through 90 days, particularly in SSI and SFQ scores.

The most pronounced improvements were observed in general symptom perception and frequency, as reflected in the Symptom Severity Index (SSI) and Symptom Frequency Questionnaire (SFQ). These instruments, though less commonly cited in CP/PPPS literature than the NIH-CPSI, provide a complementary perspective, capturing patient-centered outcomes. The consistent decline in SSI from 54 at baseline to 33 at 90 days demonstrates a nearly 40% reduction in overall symptom burden—an encouraging result for a non-pharmacological intervention. Similarly, SFQ scores showed a 35% decrease over the study period, indicating that not only were the symptoms less intense, but they also occurred less often. This dual effect—both in severity and recurrence—suggests a potentially disease-modifying role of the supplement, as opposed to transient symptomatic relief. This trend reflects a sustained and progressive reduction in perceived symptom severity, indicating that the supplement may offer cumulative benefits with continued use. Furthermore, the SFQ improvement suggests that not only did symptoms become less intense, but they also occurred less frequently.

Pain, a central and debilitating feature of CP/PPPS, showed a statistically significant reduction by day 30. The NIH-CPSI Pain Domain dropped from 10 to 6—a 40% decrease—and while there was a slight increase at day 90 (median: 7), the overall reduction remained clinically relevant. The transient nature of this change at 90 days may point toward the need for continued supplementation or a longer treatment window to sustain maximum benefit.

The NIH-CPSI LUTS domain scores also trended downward, although to a lesser extent. Baseline urinary symptoms were relatively mild to moderate (median: 7), and improvements were more modest, dropping to 6 at 90 days. This partial response suggests that CP/PPPS-related LUTS are often less responsive to therapy compared to pain symptoms and led to the observation that some improvement in urinary tract symptoms such as urgency, frequency, or weak stream, although these changes were less robust than those observed for pain.

General urinary symptom scores, as measured by IPSS, improved marginally. A reduction from 13 to 12 is not clinically dramatic but may be meaningful for patients with more severe baseline symptoms. The statistical significance at day 30 suggests a short-term benefit, though this did not progress further at 90 days. The lack of continued improvement may indicate that the supplement's impact on IPSS-specific items may be modest and possibly plateauing over time. The IPSS-QoL was stable, which may reflect a lag between symptom relief and perceived life quality enhancement.

Importantly, erectile function, as measured by the IIEF-5, remained unchanged. This result is twofold in significance: (1) the supplement does not appear to enhance sexual function, but more importantly, (2) it does not impair it—a crucial safety consideration in a male population where sexual health is often intertwined with urinary and pain symptoms.

The clinical benefits observed may stem from the synergistic effects of the supplement's components.

Curcumin has well-documented anti-inflammatory and analgesic properties. Its ability to inhibit NF-κB and reduce oxidative stress may help downregulate the chronic inflammatory state in CP/PPPS (5). Quercetin, a flavonoid found in many fruits and vegetables, has demonstrated mast cell stabilization, reduced oxidative damage, and anti-nociceptive effects in previous studies on prostatitis and interstitial cystitis (6, 7). Hyaluronic Acid and Chondroitin Sulfate contribute to mucosal protection and epithelial barrier repair, particularly in the bladder urothelium (8). Their inclusion may offer additional protection against urothelial irritation and inflammation. Collectively, these molecules may target both the immunologic and epithelial components of CP/PPPS, addressing inflammation, pain signaling, and tissue vulnerability concurrently. The urothelium and its overlying mucus layer serve as a barrier between urinary solutes (e.g., potassium, urea, ammonia) and the underlying tissue. Disruption of this barrier may contribute to various chronic urological conditions. As extensively documented in the literature (8) restoring the glycosaminoglycan (GAG) layer may support the multimodal management of complex conditions such as chronic pelvic pain (CPP) associated with primary prostate pain syndrome (PPPS). GAGs like hyaluronic acid and chondroitin sulphate may play a fundamental role in this therapeutical approach.

Our findings are consistent with existing literature, particularly studies on quercetin monotherapy for CP/PPPS. Shoskes et al. reported significant symptom reductions using quercetin in a randomized controlled trial, although limited to pain domains (6). Our study expands on this by evaluating a multi-compound approach, demonstrating benefits across multiple symptom scales.

The use of a combined supplement reflects a growing trend in urological research to approach CP/PPPS as a multifaceted syndrome rather than a single-pathology disorder. Integrative approaches targeting inflammation, barrier function, and pain signaling hold promise for improving patient outcomes in ways that monotherapies have not consistently achieved (14).

Our results are also concordant with prior clinical investigations into GAG replenishment therapy. Nickel et al. first reported the potential efficacy of pentosan polysulfate sodium (PPS) in a 2000 open-label pilot study. Treatment with PPS 100 mg three times daily over 24 weeks yielded significant improvements in symptom frequency (SFQ), severity (SSI), pain (NIH-CPSI), and overall quality of life. Although promising, this initial study was limited by its small sample size and the presence of moderate adverse events (15).

Subsequently, the same group conducted a randomized controlled trial (RCT) involving 100 men with CP/CPPS, comparing PPS 300 mg TID to placebo over 16 weeks. While the Clinical Global Improvement (CGI) outcome favored PPS (37% vs. 18%, *p* = 0.04), the difference did not reach statistical significance when compared directly to placebo (*p* = 0.107). Moreover, the reduction in NIH-CPSI scores with PPS did not significantly exceed that of the placebo group (*p* = 0.068), highlighting the inherent placebo responsiveness often seen in CP/CPPS trials. Nonetheless, the study reinforced the potential utility of PPS in a subset of patients and underscored the need for further investigation into personalized treatment strategies (16).

Indeed, the study by Manfredi et al. supports the efficacy of oral hyaluronic acid, chondroitin sulfate, curcumin, and quercetin in alleviating LUTS and bladder pain following intravesical chemotherapy, reinforcing their potential role in managing acute inflammatory symptoms. This finding aligns with and strengthens the rationale for their use in other urological conditions such as CP/PPPS (17).

Compared to these earlier studies, our investigation differs in that it evaluated a combination oral formulation with a broader mechanism of action—including antioxidant, anti-inflammatory, and barrier-restorative components. The absence of reported adverse effects in our cohort further underscores the favorable safety profile of this therapeutic approach, which may represent an important consideration in this patient population often subjected to polypharmacy and chronic symptom burden.

Gandaglia et al. reported that chronic prostatic inflammation may lead to tissue damage and continuous wound healing, thus contributing to prostatic enlargement. Chronic inflammation could enhance BPH progression which is linked to larger prostate volume, more severe urinary symptoms, and a higher risk of acute urinary retention. It may also predict poor response to treatment, highlighting the need to identify these patients for better management and tailored therapies (18). These considerations play a critical role in the management of chronic prostatitis, not only in terms of symptom control but also in preventing the progression of BPH, with Ialuril Soft Gels ® potentially contributing to a multifactorial therapeutic approach.

This study possesses several notable strengths. First, its prospective observational design, coupled with multiple time-point assessments (baseline, 30 days, and 90 days), enabled temporal evaluation of symptom trajectories and treatment response. The use of standardized, validated patient-reported outcome measures (PROMs)—including the NIH-CPSI, IPSS, and IIEF-5—ensured a consistent and clinically meaningful evaluation of symptom severity, urinary function, and QoL. Additionally, the study was conducted in a real-world outpatient setting, enhancing the generalizability of findings to typical clinical practice for chronic prostatitis/chronic pelvic pain syndrome (CP/CPPS). Importantly, the intervention demonstrated an excellent safety profile, with no adverse events reported during the study period, further supporting its tolerability in this patient population.

However, several limitations must be acknowledged. The relatively small sample size restricted the statistical power of the study and limits the generalizability of the findings to broader populations. The absence of a placebo or control group precludes definitive conclusions regarding treatment efficacy and makes it difficult to account for potential placebo effects. Moreover, the short duration of follow-up (90 days) limits insights into the long-term durability of the observed clinical benefits. The study also lacked objective biomarker, laboratory, or imaging data to corroborate subjective symptom changes with physiologic or structural correlates.

Future research should aim to address these limitations through larger, randomized controlled trials (RCTs) with longer follow-up durations and the inclusion of biomolecular or imaging endpoints. Such studies would be instrumental in validating the observed effects and elucidating the potential mechanisms of action underlying this multimodal nutraceutical approach.

## Conclusion

In this prospective observational study, the administration of Ialuril Soft Gels ® containing curcumin, quercetin, hyaluronic acid, and chondroitin sulfate resulted in meaningful clinical improvements in men with Chronic Prostatitis/Primary Prostate Pain Syndrome. Statistically and clinically significant reductions were observed in symptom severity and frequency, with additional moderate improvements in pain and urinary symptoms. The supplement was well tolerated, with no adverse events, and had a neutral effect on erectile function.

While these findings are promising and support the role of multi-target supplements in the management of CP/PPPS, larger, controlled trials are warranted to further establish efficacy and optimize treatment protocols. For now, Ialuril Soft Gels ® appears to be a safe and potentially effective option in the adjunctive management of this challenging urologic condition and it may play a role in preventing the progression of benign prostatic hyperplasia.

## Data Availability

The raw data supporting the conclusions of this article will be made available by the authors, without undue reservation.
